# Involvement of apoptotic pathways in docosahexaenoic acid-induced benefit in prostate cancer: Pathway-focused gene expression analysis using RT^2^ Profile PCR Array System

**DOI:** 10.1186/s12944-017-0442-5

**Published:** 2017-03-23

**Authors:** Yanan Sun, Xiaopeng Jia, Lianguo Hou, Xing Liu, Qiang Gao

**Affiliations:** 1 Department of Obstetrics and Gynecology, Bethune International Peace Hospital of the People’s Liberation Army, Shijiazhuang, 050082 China; 2 Department of Urology, The Third Hospital of Hebei Medical University, Shijiazhuang, 050051 China; 3 Department of Biochemistry and Molecular Biology, Hebei Medical University, Shijiazhuang, 050017 China; 4 Department of Orthopaedic Trauma, Section II, The Third Hospital of Shijiazhuang City, Shijiazhuang, Hebei 050011 China; 5 Department of Nutrition and Food hygiene, Hebei Medical University, Shijiazhuang, 050017 China

**Keywords:** Human prostate carcinoma cell, DU145 cells, Docosahexaenoic acid (DHA), RT^2^ profile PCR array system

## Abstract

**Background:**

Present study aimed to better understand the potential apoptotic pathways that involved in docosahexaenoic acid (DHA)-induced apoptosis of prostate cancer cells.

**Methods:**

Human prostate cancer DU145 cells were treated with different concentrations of fish oil, omega-3 PUFA (DHA, and Eicosapentaenoic acid, EPA), or omega-6 PUFA (Arachidonic acid, AA). Cell viability and apoptosis were evaluated by MTT assay and Hoechst staining. Pathway-focused gene expression profiling of DU145 cells was analyzed with the RT^2^ Profile PCR Array System. The results were verified by real time quantitative polymerase chain reaction (RT-qPCR).

**Results:**

AA exposure showed no obvious effect on viability of DU145 cells. However, exposure with fish oil, EPA, or DHA for 24 h significantly affected cell viability. The growth inhibition of DHA was more pronounced than that of EPA and showed a time-dependent increase. DHA exposure caused typical apoptotic characteristics. Ten genes were more expressed, while 5 genes were less expressed following DHA exposure. RT-qPCR confirmed the time dependent effect of DHA on the expression of these differentially expressed genes. KEGG pathway analysis showed that DHA may induce the apoptosis of cancer cells preferentially through mediating P53, MAPK, TNF, PI3K/AKT, and NF-κB signaling pathways.

**Conclusion:**

Our study demonstrated the beneficial action of DHA on human prostate carcinoma cell line DU145. The pro-apoptotic effect of DHA on DU145 cells may involve mediation various pathways, especially P53, MAPK, TNF, PI3K/AKT, and NF-κB signaling pathways. Molecular mechanisms of DHA on apoptosis of cancer cells still need to be further clarified.

**Electronic supplementary material:**

The online version of this article (doi:10.1186/s12944-017-0442-5) contains supplementary material, which is available to authorized users.

## Background

Prostate cancer is one of the most commonly diagnosed cancers in elderly men, with approximately one million new cases diagnosed per year worldwide [1]. The incidence of prostate cancer in Asian populations has long been established to be lower than that in Western populations. However, the prevalence of the disease has shown a rapid increase in Asian countries, including China [2, 3]. More and more cases of prostate cancer have been diagnosed in China in recent years [4]. In Shanghai alone, the incidence of prostate cancer has increased from 1.6 to 7.7 per 10,000 person (4.8 folds) during 1973 to 2000 [5]. One explanation for this rapid change is the westernization of dietary life style [6]. Compared to Eastern counterpart, the diet of Western population is generally characterized by higher levels of saturated fats that may contribute to the development of prostate cancer [7]. Daily intake of the individual fatty acids such as omega-3 polyunsaturated fatty acids (PUFA) has been reported to be comparatively low in most Westernized countries [8]. Dietary factors, including the consumption of unsaturated fat, may reduce the risk or progression of prostate cancer [9].

Certain omega-3 PUFA, including docosahexaenoic acid (DHA), have been well recognized to exert beneficial effects in prevention of a series of chronic diseases, including cancer [10]. Dietary fat fish, fish oils and their active omega-3 PUFAs constituents have been associated with the reduced risk of prostate cancer [8, 11, 12]. Various pathways have been reported to involve in the antiproliferative effects of omega-3 PUFAs in prostate cancer [13–15]. It has been suggested that omega-3 PUFAs may inhibit the incidence and development of prostate cancer through various mechanisms [16, 17]. The regulation of the apoptotic pathways is one of the potential mechanisms that responsible for the beneficial effects of dietary omega-3 PUFA [18]. The increased apoptosis was observed following the treatment with a fish oil diet or DHA, with the possible induction of both the intrinsic and extrinsic apoptotic pathways [19]. The pro-apoptotic effect of DHA has been reported to involve modifications of the expression and activity of different intracellular apoptotic pathways [20, 21]. Therefore, to better understand the potential apoptotic pathways involved in DHA-induced apoptosis of prostate cancer cells, pathway-focused gene expression analysis was performed in our study using RT^2^ Profile PCR Arrays.

## Methods

### Cell culture and treatment

The human prostate carcinoma cell line DU145 was purchased from the Shanghai cell bank of Chinese Academy of Sciences (Shanghai, China). DU145 cells were cultured in Dulbecco’s modified eagle medium (DMEM, Gibico, Carlsbad, CA, USA) supplemented with 10% fetal bovine serum (FBS, Hangzhou Sijiqing Company, China), 100 U/mL penicillin and 0.1 mg/mL streptomycin. Cells were incubated in a 5% CO_2_ incubator (Sanyo Electric Biomedical CO., LTD., Japan) at 37 °C. The media were refreshed every 2–3 days. Fish oil (Shanghai Haling biotechnology Co., Ltd., Shanghai, China), DHA, eicosapentaenoic acid (EPA), and arachidonic acid (AA, 50 mmol/L, Sigma) were prepared using NaOH and were dissolved in BSA (0.01 mol/L PBS, 1:9). Media with and without BSA were used as BSA and blank controls.

### MTT assay

Cell viability was measured using an MTT kit (Sigma). DU145 cells (200 μL, 2 × 10^4^/mL) were seeded in 96-well plates and synchronized by serum starvation in DMEM containing 1% FBS for 24 h. The cells were then incubated with fish oil at a final concentration of 50, 150, 250, and 500 μmol/L, or with AA, EPA, and DHA at a final concentration of 10, 25, 50, and 100 μmol/L for 24 h, respectively. Control cells were treated with BSA (BSA group) or without any treatment (Blank control). MTT (5 mg/mL, 20 μL) was added to each well and incubated for 4 h. The supernatants were removed and dimethyl sulfoxide (150 μL, Sigma) was added. The absorbance was measured at 490 nm using a DH5031 enzyme-linked immunometric meter (Nanjing Medical Instrument Factory, China).

### Hoechst staining

Cell apoptosis was determined by Hoechst staining. DU145 cells (8 × 10^4^) were plated in a 24-well plate and incubated with DHA for 24 h. Cells were then fixed with acetone for 30 min, followed by staining with Hoechst 33258 (Beyotime Institute of Biotechnology, Jiangsu, China) for 15 min. Apoptosis of cells was assayed under a fluorescent microscope (Olympus, Tokyo, Japan).

### RT2 Profile PCR Arrays

DU145 cells treated with DHA were then underwent pathway-focused gene expression analyses. Total RNA was extracted with Trizol and was quantified by NanoDrop ND-1000 Spectrophotometer (NanoDrop Technologies). First-strand cDNA was synthesized using a RT^2^ first-strand kit (SABiosciences, Frederick, MD, USA) according to the manufacturer’s instructions. Pathway-focused gene expression analyses were performed using RT2 Profile PCR Arrays (PAHS-012Z, Qiagen, USA). The expressions of 84 key genes involved in apoptosis-related pathways were measured.

### Real time quantitative polymerase chain reaction (RT-qPCR)

To validate the effect of DHA on expression of genes that mediating the activity of apoptosis-related pathways, RT-qPCR analysis was further performed. After treatment of DU145 cells with different concentrations of DHA for 24 h, total RNA was extracted and reverse transcribed to cDNA. RT-qPCR was conducted using a SYBR Green quantitative RT-PCR kit (Invitrogen, Carlsbad, California). PCR program was 50 °C and 95 °C each for 2 min followed by 40 cycles at 95 °C for 15 s and 52 °C for 30 s. The primers used are summarized in Table 1. Data were analyzed by 2^−ΔΔCt^ method using TBP as a reference gene.

**Table 1:** Primes used for RT-qPCR

Gene	Prime	Length (bp)
Caspase 3	Sense: 5’-TCTGGTTTTCGGTGGGTGT-3’	287
	Antisense:5’-TGAGGTTTGCTGCATCGACAT-3’	
Caspase 1	Sense: 5’-ACATCCCACAATGGGCTCTG-3’	233
	Antisense:5’-TTCACTTCCTGCCCACAGAC-3’	
Caspase 9	Sense: 5’-CAGGCCCCATATGATCGAGG-3’	194
	Antisense:5’-TCGACAACTTTGCTGCTTGC-3’	
Caspase 12	Sense: 5’-ATCCAACGGTGTTCTGGTCC-3’	360
	Antisense:5’-TCTCGCATCCCCAAAAGGTC-3’	
TNF	Sense: 5’-CTGGGCAGGTCTACTTTGGG-3’	272
	Antisense:5’-CTGGAGGCCCCAGTTTGAAT-3’	
TP53	Sense: 5’-ACCTATGGAAACTACTTCCTGAAA-3’	500
	Antisense:5’-ACCATCGCTATCTGAGCAGC-3’	
BCL2	Sense: 5’-TTTGTGGAACTGTACGGCCC-3’	451
	Antisense: 5’-CGGTGCTTGGCAATTAGTGG-3’	
CIDEA	Sense: 5’-GTGCAGGCAGACAGACCTCC-3’	487
	Antisense: 5’-CTTCACGTTAAGGCAGCCGA-3’	
DFFA	Sense: 5’-TACGTCAGAGTTGTGCCACC-3’	312
	Antisense:5’-TGGTAACCAACTCCAAATCCTGA-3’	
AIFM1	Sense: 5’-GTTCCAGCGATGGCATGTTC-3’	230
	Antisense: 5’-ACGCGGCCTTTTTCTGTTTC-3’	
AKT1	Sense: 5’-CAGGAGGTTTTTGGGCTTGC-3’	418
	Antisense: 5’-TGTACTCCCCTCGTTTGTGC-3’	
BID	Sense: 5’-CATAAGGAGGAAGCGGGTAGTC-3’	174
	Antisense: 5’-ACCGTTGTTGACCTCACAGT-3’	
AKT1	Sense: 5’-CAGCCTCCCTCCGAGTTTG-3’	446
	Antisense: 5’-ACCTGGTTTAGCACTGAGCG-3’	
XIAP	Sense: 5’-ATTTCCAGATTGGGGCTCGG-3’	449
	Antisense:5’-TTTGTAGACTGCGTGGCACT-3’	
TBP	Sense: 5’-CAGGGGTTCAGTGAGGTCG-3’	107
	Antisense: 5’-ACCCTGGGTCACTGCAAAG-3’	

### Statistical analysis

Data were expressed as mean ± standard deviation (SD). Statistical significance was analyzed using ANOVA followed by least significant difference test or t test. All statistical analysis was performed with SPSS, version17.0 (SPSS Inc., Chicago, IL, USA). A p value of less than 0.05 was considered as statistically significant.

## Results

### Effect of fish oil, AA, EPA, or DHA on DU145 cell viability

**Fig. 1:**
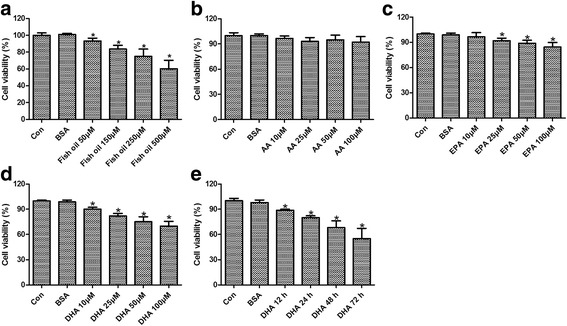
Viability of human prostate cancer DU145 cells as determined by MTT assay. The dose dependent effect of fish oil (**a**), AA (**b**), EPA (**c**), and DHA (**d**) on viability of DU145 cells. The time-dependent effect of DHA on cell viability (**e**). ^*^ *p* < 0.05 vs the control groups

The viability of DU145 cells was determined by MTT assay. As shown in Fig. 1a, after exposure with different concentrations of fish oil for 24 h, viability of DU145 cells was significantly affected and showed a dose-dependent decrease. Therefore, the effect of dose dependent effect of AA, EPA, or DHA on viability of DU145 cells was further investigated. AA exposure showed no obvious effect on viability of DU145 cells (Fig. 1b). By contrast, exposure of DU145 cells to EPA and DHA significantly inhibited cell viability in a dose dependent manner (Fig. 1c and d). The growth inhibition of DHA was more pronounced than that of EPA. Therefore, DHA was used in the following study. Time dependent effect of DHA exposure on the viability of DU145 cells was further evaluated with 50 μmol/L DHA. As shown in Fig. 1e, growth inhibition-induced by DHA was enhanced with the increased time.

### Effect of DHA on DU145 cell apoptosis

**Fig. 2:**
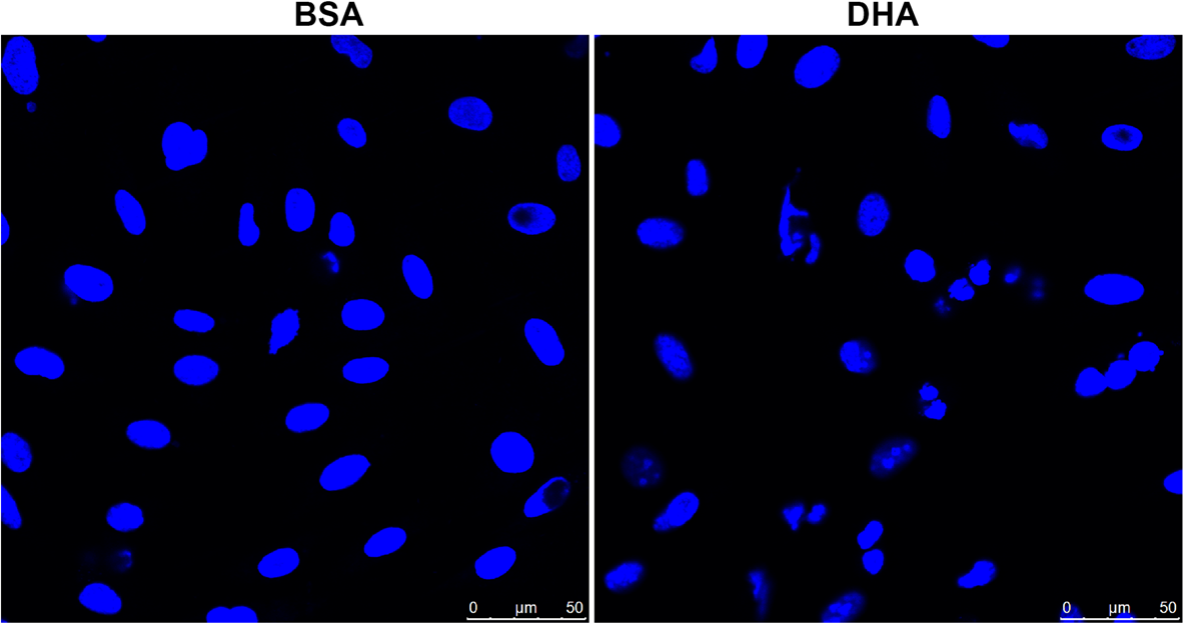
Effect of DHA exposure on apoptosis of DU145 cells assessed by Hoechst 33258 staining

Apoptosis of DU145 cells was evaluated following the exposure of cells with DHA (50 μmol/L) for 24 h. Morphological analysis using Hoechst 33258 stain showed typical apoptotic characteristics of condensation and segmentation of the nucleus after DHA exposure (Fig. 2).

### Effect of DHA on apoptosis pathway-focused gene expression profiling

**Fig. 3:**
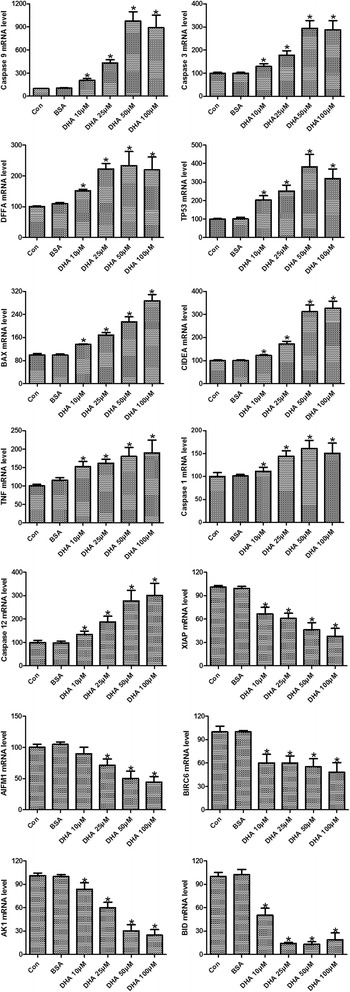
RT-qPCR analysis of the time-dependent effect of DHA exposure on differentially expressed genes identified by pathway-focused gene expression analysis. ^*^ *p* < 0.05 vs the BSA group

To gain insight of genes involving in DHA-induced apoptosis, pathway-focused gene expression profiling of DU145 cells was analyzed with the RT^2^ Profile PCR Array System. The differentially expressed genes were identified by fold changes greater than 2-folds. DU145 cells exposed to DHA showed an obviously different gene expression pattern when compared with the control (Additional file 1: Table S1). Ten genes were identified to be upregulated greater than 2-folds after DHA exposure, including three apoptosis-related cysteine peptidases, caspase 1, 3 and 9, pro-apoptotic protein BAX, cell death-inducing factors CIDEA and DFFA, tumor necrosis related factors TNFRSF1A, LTA and TNF, tumor suppressor gene TP53 (Table 2). Meanwhile, 5 genes were found to be downregulated at least 2-folds, they were X-linked apoptosis inhibitor protein XIAP, mitochondrion-associated apoptosis inducing factor AIFM1, protein kinase AKT1, BH3-containing proteins BID and BIRC6. To confirm the findings of our pathway-focused gene expression analysis, the time-dependent effect of DHA on some of the differentially expressed genes were further assessed by RT-qPCR. As shown in Fig. 3, DHA treatment for 24 h significantly upregulated the expressions of caspase 1, 3 and 9, BAX, CIDEA, DFFA, TNF and TP53, while downregulated the expressions of XIAP, AIFM1, AKT1, BID and BIRC6 (*p* < 0.05). DHA treatment of DU145 cells showed a time dependent effect on the expressions of Caspase 3 and 9, BAX, CIDEA, TNF, XIAP, AIFM1 and BID (*p* < 0.05). These up- and down-regulated genes were further subjected to KEGG pathway analyses. KEGG pathway annotation showed that the targeted genes were highly enriched in several pathways, mainly involved in the P53 signaling pathway, MAPK signaling pathway, TNF signaling pathway, PI3K/AKT signaling pathway, and NF-κB signaling pathway (Table 3).

**Table 2:** Effect of DHA on apoptosis pathway-focused gene expression profiling of DU145 cells

Refseq	Symbol	Description	Folds
NM_001229	CASPASE 9	Caspase 9, apoptosis-related cysteine peptidase	12.10
NM_004346	CASPASE 3	Caspase 3, apoptosis-related cysteine peptidase	4.88
NM_004401	DFFA	DNA fragmentation factor, 45 kDa, alpha polypeptide	3.21
NM_000546	TP53	Tumor protein p53	2.97
NM_004324	BAX	BCL2-associated X protein	2.93
NM_001279	CIDEA	Cell death-inducing DFFA-like effector a	2.34
NM_000594	TNF	Tumor necrosis factor	2.24
NM_001065	TNFRSF1A	Tumor necrosis factor receptor superfamily, member 1A	2.14
NM_033292	CASPASE 1	Caspase 1, apoptosis-related cysteine peptidase	2.06
NM_000595	LTA	Lymphotoxin alpha (TNF superfamily, member 1)	2.04
NM_001167	XIAP	X-linked inhibitor of apoptosis	0.49
NM_004208	AIFM1	Apoptosis-inducing factor, mitochondrion-associated, 1	0.45
NM_016252	BIRC6	Baculoviral IAP repeat containing 6	0.32
NM_005163	AKT1	V-akt murine thymoma viral oncogene homolog 1	0.21
NM_001196	BID	BH3 interacting domain death agonist	0.11

**Table 3:** Pathway enrich of apoptotic genes

Components	Gene count	Representative genes
P53 signaling pathway	6	Caspase 3, Caspase 9, TP53, Bax, AKT1, BID
MAPK signaling pathway	5	Caspase 3, TP53, TNF, TNFRSF1A, AKT1
TNF signaling pathway	4	Caspase 3, TNF, XIAP, AKT1
PI3K/AKT signaling pathway	2	Caspase 9, TP53
NF-κB signaling pathway	2	TNF, XIAP

## Discussion

Dietary fat consumption is a critical risk factor for development and progression of prostate cancer [8, 22]. Omega-3 PUFA, particularly DHA, have shown important inverse associations with tumorigenesis and progression, with the inhibited cell proliferation and reduced incidence and progression of the tumors [23, 24]. Alteration in apoptosis may play a crucial role in this procedure [20, 25, 26]. However, the potential mechanisms have not been fully elucidated [27]. The critical analysis of PUFA action on potential apoptotic pathways have been carried out in this study. The findings of our study showed that DHA may modulate the apoptosis of prostate cells through different pathways. DHA exposure upregulated the gene expressions of Caspase 9, Caspase 3, DFFA, TP53, BAX, CIDEA, TNF, TNFRSF1A, Caspase 1, and LTA, while downregualted XIAP, AIFM1, BIRC6, AKT1, and BID expressions. KEGG pathway annotation showed that DHA exposure mainly involved in the p53 pathway, TNF signaling pathway, AKT-related signaling pathway, and mitochondrial-related pathway, suggesting the modulation of the both extrinsic and intrinsic apoptotic pathways by DHA. Some of such pathways have been reported in DHA-induced pro-apoptosis of cancer cells [14, 28–30].

Previous studies indicated that increased dietary intakes of omega-6 PUFA like AA may worsen the development of prostate cancer. By contrast, intakes of omega-3 PUFA, such as EPA and DHA, may improve the development of prostate cancer [12]. A high dietary ratio of n-6/n-3 fatty acids has been reported to increase the risk of prostate cancer [7]. In our study, AA exposure caused no obvious inhibition of proliferation of DU145 cells. This was quietly different from the results reported by Meng et al., which indicated that AA supplementation (25, 50, 75, 100, 125, 150, 175 μmol/L) resulted in significantly decreased viability of human prostate cancer PC-3 cells [22]. This conflicting result may be partially explained by the different concentration gradients and prostate cell lines used. However, exposure of DU145 cells to the EPA or DHA in our study markedly reduced the cell viability, and this effect showed a dose-dependent pattern. The more pronounced growth inhibition of DHA was observed. Similar results have been reported, which indicated that DHA was a tumor reducing omega-3 PUFA for both androgen-dependent and -independent prostate cancer cell lines (LNCaP cells PC-3 cells) [22, 31, 32]. However, the study by Chung et al. indicated that DHA treatment showed litter response in androgen-dependent prostate cancer LNCaP cells [33]. Schley et al. have reported that DHA was more potent in growth inhibition of human breast cancer MDA-MB-231 cells than that of EPA [24]. While Liu and colleagues showed more efficacious growth inhibition of EPA than that of DHA [13]. The possible explanations for this disparity could be the different doses and cell lines used in these studies. Despite of these conflicting results, our results did show a pronounced growth inhibitory effect of omega-3 PUFA, EPA and DHA, on androgen-independent, hormone refractory prostate cancer DU145 cells. DHA exposure caused a time-dependent growth inhibition on DU145 cells. Furthermore, apoptosis of DU145 cells was also found to be significantly enhanced following DHA treatment. All these results showed evidence suggested that omega-3 PUFA, particularly DHA, may exert beneficial effect on prostate cancer through inhibiting proliferation and promoting apoptosis of cancer cells.

The anticancer activity of omega 3-PUFA (DHA) has been suggested to be due to apoptosis induction of fatty acid [25, 34–36]. Different apoptotic pathways have been reported to involve in the anticancer actions of omega-3 fatty acids (DHA). The p53-mediated signaling pathways have been identified to involve in DHA-induced apoptosis and autophagy in cancer cells harboring wild-type p53 [28]. In prostate cancer cells harboring mutant p53, however, mitochondrial ROS-mediated Akt-mTOR signaling was found to induce apoptosis and autophagy [29]. DHA has also been shown to induce apoptosis through inhibiting the Akt/NF-κB pathway and by modulating the PI3K/AKT/p38 MAPK pathway [14, 24, 37, 38]. The suppression of genes involved in PDK1/Akt/Bad signaling and NF-kB pathways has also been suggested to contribute to DHA-induced apoptosis [30, 39]. However, the pro-apoptotic molecular mechanisms of DHA have not been well established. Therefore, in the present study, the apoptosis-related genes that abnormally expressed in DHA-treated prostate cancer DU145 cells were screened using the specifically designed RT^2^ Profile PCR arrays. Among the 84 key genes involved in mediating apoptosis-related pathways, 10 genes were more expressed in DHA-exposed DU145 cells than control, while 5 genes were less expressed following DHA exposure (more than two-fold change). To confirm the accuracy of the present results, RT-qPCR was performed to validate some of the differently expressed genes in a time-dependent manner. The results showed that the expressions of these differently expressed genes were consistent with the data from microarray. KEGG pathway analysis of our study showed that DHA exposure may induce the apoptosis of cancer cells preferentially through mediating P53, MAPK, TNF, PI3K/AKT, and NF-κB signaling pathways, partially corroborating the results of previous studies. However, future studies are still needed to further clarify the molecular mechanisms of DHA-induced proapoptosis of cancer cells.

## Conclusions

In conclusion, our study demonstrated the beneficial action of DHA on human prostate carcinoma cell line DU145. The pro-apoptotic effect of DHA on DU145 cells may involve mediating P53 signaling pathway, MAPK signaling pathway, TNF signaling pathway, PI3K/AKT signaling pathway, and NF-κB signaling pathway. Molecular mechanisms of DHA on apoptosis of cancer cells still need further clarify.

## Additional file

Additional file 1: Table S1. Apoptosis pathway-focused gene expression profiling of DU145 cells following DHA exposure. (DOCX 21 kb)

## Abbreviations

AA: Arachidonic acid
DHA: Docosahexaenoic acid
EPA: Eicosapentaenoic acid
PUFA: Polyunsaturated fatty acids
RT-qPCR: Real time quantitative polymerase chain reaction
