# Supplementary material for: Involvement of apoptotic pathways in docosahexaenoic acid-induced benefit in prostate cancer: Pathway-focused gene expression analysis using RT2 Profile PCR Array System
Source: Lipids Health Dis. 2017 Mar 23;16:59. doi: 10.1186/s12944-017-0442-5 (PMC5363041; doi:10.1186/s12944-017-0442-5)
Supplement: Additional file 1: Table S1. — Apoptosis pathway-focused gene expression profiling of DU145 cells following DHA exposure. (DOCX 21 kb) [file 12944_2017_442_MOESM1_ESM.docx]

**Supplemental Table 1** Apoptosis pathway-focused gene expression profiling of DU145 cells following DHA exposure

| **Refseq** | **Symbol** | **Description** | **Fold change** |
| --- | --- | --- | --- |
| NM_005157 | ABL1 | C-abl oncogene 1, non-receptor tyrosine kinase | 1.95 |
| NM_004208 | AIFM1 | Apoptosis-inducing factor, mitochondrion-associated, 1 | 0.45 |
| NM_005163 | AKT1 | V-akt murine thymoma viral oncogene homolog 1 | 0.21 |
| NM_001160 | APAF1 | Apoptotic peptidase activating factor 1 | 0.93 |
| NM_004322 | BAD | BCL2-associated agonist of cell death | 0.55 |
| NM_004323 | BAG1 | BCL2-associated athanogene | 0.83 |
| NM_004281 | BAG3 | BCL2-associated athanogene 3 | 1.02 |
| NM_001188 | BAK1 | BCL2-antagonist/killer 1 | 1.09 |
| NM_004324 | BAX | BCL2-associated X protein | 2.93 |
| NM_003921 | BCL10 | B-cell CLL/lymphoma 10 | 0.98 |
| NM_000633 | BCL2 | B-cell CLL/lymphoma 2 | 1.33 |
| NM_004049 | BCL2A1 | BCL2-related protein A1 | 0.67 |
| NM_138578 | BCL2L1 | BCL2-like 1 | 0.52 |
| NM_020396 | BCL2L10 | BCL2-like 10 (apoptosis facilitator) | 0.69 |
| NM_006538 | BCL2L11 | BCL2-like 11 (apoptosis facilitator) | 0.56 |
| NM_004050 | BCL2L2 | BCL2-like 2 | 1.76 |
| NM_016561 | BFAR | Bifunctional apoptosis regulator | 1.31 |
| NM_001196 | BID | BH3 interacting domain death agonist | 0.11 |
| NM_001197 | BIK | BCL2-interacting killer (apoptosis-inducing) | 0.71 |
| NM_001166 | BIRC2 | Baculoviral IAP repeat containing 2 | 1.19 |
| NM_001165 | BIRC3 | Baculoviral IAP repeat containing 3 | 0.64 |
| NM_001168 | BIRC5 | Baculoviral IAP repeat containing 5 | 0.96 |
| NM_016252 | BIRC6 | Baculoviral IAP repeat containing 6 | 0.32 |
| NM_004330 | BNIP2 | BCL2/adenovirus E1B 19kDa interacting protein 2 | 1.31 |
| NM_004052 | BNIP3 | BCL2/adenovirus E1B 19kDa interacting protein 3 | 1.74 |
| NM_004331 | BNIP3L | BCL2/adenovirus E1B 19kDa interacting protein 3-like | 1.09 |
| NM_004333 | BRAF | V-raf murine sarcoma viral oncogene homolog B1 | 1.60 |
| NM_033292 | CASP1 | Caspase 1, apoptosis-related cysteine peptidase | 2.06 |
| NM_001230 | CASP10 | Caspase 10, apoptosis-related cysteine peptidase | 0.90 |
| NM_012114 | CASP14 | Caspase 14, apoptosis-related cysteine peptidase | 0.86 |
| NM_032982 | CASP2 | Caspase 2, apoptosis-related cysteine peptidase | 0.69 |
| NM_004346 | CASP3 | Caspase 3, apoptosis-related cysteine peptidase | 4.88 |
| NM_001225 | CASP4 | Caspase 4, apoptosis-related cysteine peptidase | 0.58 |
| NM_004347 | CASP5 | Caspase 5, apoptosis-related cysteine peptidase | 1.62 |
| NM_032992 | CASP6 | Caspase 6, apoptosis-related cysteine peptidase | 0.90 |
| NM_001227 | CASP7 | Caspase 7, apoptosis-related cysteine peptidase | 1.02 |
| NM_001228 | CASP8 | Caspase 8, apoptosis-related cysteine peptidase | 0.85 |
| NM_001229 | CASP9 | Caspase 9, apoptosis-related cysteine peptidase | 12.10 |
| NM_001242 | CD27 | CD27 molecule | 0.98 |
| NM_001250 | CD40 | CD40 molecule, TNF receptor superfamily member 5 | 0.50 |
| NM_000074 | CD40LG | CD40 ligand | 0.62 |
| NM_001252 | CD70 | CD70 molecule | 0.99 |
| NM_003879 | CFLAR | CASP8 and FADD-like apoptosis regulator | 0.57 |
| NM_001279 | CIDEA | Cell death-inducing DFFA-like effector a | 2.34 |
| NM_014430 | CIDEB | Cell death-inducing DFFA-like effector b | 0.99 |
| NM_003805 | CRADD | CASP2 and RIPK1 domain containing adaptor with death domain | 1.10 |
| NM_018947 | CYCS | Cytochrome c, somatic | 1.13 |
| NM_004938 | DAPK1 | Death-associated protein kinase 1 | 1.12 |
| NM_004401 | DFFA | DNA fragmentation factor, 45kDa, alpha polypeptide | 3.21 |
| NM_019887 | DIABLO | Diablo, IAP-binding mitochondrial protein | 0.94 |
| NM_003824 | FADD | Fas (TNFRSF6)-associated via death domain | 1.65 |
| NM_000043 | FAS | Fas (TNF receptor superfamily, member 6) | 0.98 |
| NM_000639 | FASLG | Fas ligand (TNF superfamily, member 6) | 1.21 |
| NM_001924 | GADD45A | Growth arrest and DNA-damage-inducible, alpha | 1.34 |
| NM_003806 | HRK | Harakiri, BCL2 interacting protein (contains only BH3 domain) | 0.92 |
| NM_000875 | IGF1R | Insulin-like growth factor 1 receptor | 1.37 |
| NM_000572 | IL10 | Interleukin 10 | 0.78 |
| NM_000595 | LTA | Lymphotoxin alpha (TNF superfamily, member 1) | 2.04 |
| NM_002342 | LTBR | Lymphotoxin beta receptor (TNFR superfamily, member 3) | 1.44 |
| NM_021960 | MCL1 | Myeloid cell leukemia sequence 1 (BCL2-related) | 0.89 |
| NM_004536 | NAIP | NLR family, apoptosis inhibitory protein | 0.86 |
| NM_003998 | NFKB1 | Nuclear factor of kappa light polypeptide gene enhancer in B-cells 1 | 0.69 |
| NM_006092 | NOD1 | Nucleotide-binding oligomerization domain containing 1 | 0.78 |
| NM_003946 | NOL3 | Nucleolar protein 3 (apoptosis repressor with CARD domain) | 0.71 |
| NM_013258 | PYCARD | PYD and CARD domain containing | 0.57 |
| NM_003821 | RIPK2 | Receptor-interacting serine-threonine kinase 2 | 0.85 |
| NM_000594 | TNF | Tumor necrosis factor | 2.24 |
| NM_003844 | TNFRSF10A | Tumor necrosis factor receptor superfamily, member 10a | 0.65 |
| NM_003842 | TNFRSF10B | Tumor necrosis factor receptor superfamily, member 10b | 0.85 |
| NM_002546 | TNFRSF11B | Tumor necrosis factor receptor superfamily, member 11b | 1.07 |
| NM_001065 | TNFRSF1A | Tumor necrosis factor receptor superfamily, member 1A | 2.14 |
| NM_001066 | TNFRSF1B | Tumor necrosis factor receptor superfamily, member 1B | 1.08 |
| NM_014452 | TNFRSF21 | Tumor necrosis factor receptor superfamily, member 21 | 1.75 |
| NM_003790 | TNFRSF25 | Tumor necrosis factor receptor superfamily, member 25 | 0.85 |
| NM_001561 | TNFRSF9 | Tumor necrosis factor receptor superfamily, member 9 | 1.18 |
| NM_003810 | TNFSF10 | Tumor necrosis factor (ligand) superfamily, member 10 | 0.70 |
| NM_001244 | TNFSF8 | Tumor necrosis factor (ligand) superfamily, member 8 | 0.75 |
| NM_000546 | TP53 | Tumor protein p53 | 2.97 |
| NM_005426 | TP53BP2 | Tumor protein p53 binding protein, 2 | 0.96 |
| NM_005427 | TP73 | Tumor protein p73 | 0.96 |
| NM_003789 | TRADD | TNFRSF1A-associated via death domain | 0.79 |
| NM_021138 | TRAF2 | TNF receptor-associated factor 2 | 0.96 |
| NM_003300 | TRAF3 | TNF receptor-associated factor 3 | 0.92 |
| NM_001167 | XIAP | X-linked inhibitor of apoptosis | 0.49 |
| NM_001101 | ACTB | Actin, beta | 0.86 |
| NM_004048 | B2M | Beta-2-microglobulin | 0.51 |
| NM_002046 | GAPDH | Glyceraldehyde-3-phosphate dehydrogenase | 1.27 |
| NM_000194 | HPRT1 | Hypoxanthine phosphoribosyltransferase 1 | 1.22 |
| NM_001002 | RPLP0 | Ribosomal protein, large, P0 | 1.22 |
| SA_00105 | HGDC | Human Genomic DNA Contamination | 0.96 |
| SA_00104 | RTC | Reverse Transcription Control | 0.93 |
| SA_00104 | RTC | Reverse Transcription Control | 0.92 |
| SA_00104 | RTC | Reverse Transcription Control | 0.98 |
| SA_00103 | PPC | Positive PCR Control | 1.17 |
| SA_00103 | PPC | Positive PCR Control | 0.94 |
| SA_00103 | PPC | Positive PCR Control | 0.67 |
